# Diagnostic performance of combined canine and second molar maturity for identification of growth phase

**DOI:** 10.1186/2196-1042-14-1

**Published:** 2013-05-20

**Authors:** Giuseppe Perinetti, Roberto Di Lenarda, Luca Contardo

**Affiliations:** 1 Department of Medical, Surgical and Health Sciences, University of Trieste, Trieste, Italy

## Abstract

**Background:**

The objective of this research is to analyze the diagnostic performance of the circumpubertal dental maturation stages of the mandibular canine and second molar, as individual teeth and in combination, for the identification of growth phase.

**Methods:**

A total of 300 healthy subjects, 192 females and 108 males, were enrolled in the study (mean age, 11.4 ± 2.4 years; range, 6.8 to 17.1 years). Dental maturity was assessed through the calcification stages from panoramic radiographs of the mandibular canine and second molar. Determination of growth phase (as pre-pubertal, pubertal, and post-pubertal) was carried out according to the cervical vertebral maturation method. The diagnostic performances of the dental maturation stages, as both individual teeth and in combination, for the identification of the growth phase were evaluated using positive likelihood ratios (LHRs), with a threshold of ≥10 for satisfactory performance.

**Results:**

For the individual dental maturation stages, most of these positive LHRs were ≤1.6, with values ≥10 seen only for the identification of the pre-pubertal growth phase for canine stage F and second molar stages D and E, and for the post-pubertal growth phase for second molar stage H. All of the combined dental maturation stages yielded positive LHRs up to 2.6.

**Conclusions:**

Dental maturation of either individual or combined teeth has little role in the identification of the pubertal growth spurt and should not be used to assess timing for treatments that are required to be performed at this growth phase.

## Background

The identification of skeletal maturity, with particular reference to the onset of the pubertal growth spurt, has major clinical implications when dealing with orthodontic treatment in growing subjects, especially when dentoskeletal disharmonies are present [1,2]. The correct identification of the different growth phases, i.e., pre-pubertal, pubertal, and post-pubertal, on an individual basis represents a crucial issue in orthodontic diagnosis and treatment planning [2]. For this reason, and because chronological age [3] and tooth emergence [4,5] have been shown to be unreliable indicators of the onset of the pubertal growth spurt, over the last five decades, several other radiographical [2,6,7] and biochemical [8,9] indicators of the growth phases have been investigated.

As one of these indicators, dental maturity detected through radiographic methods has been shown to be highly related to skeletal maturity [10-14], especially for the mandibular canines [10,12] and the second molars [14]. In spite of these high correlations, the reliability, or diagnostic performance, of dental maturity for use as an assessment of the different growth phases appears to be low, with poor clinical meaning, which is limited mainly to the identification of the pre-pubertal growth phase [15]. However, all of these investigations were focused on the maturational stages of individual teeth, including the only diagnostic performance study [15]. Interestingly, the mandibular canine up to stage E and the second molar from stage H (according to the staging described by Demirjian et al. [16]) have been shown to have satisfactorily diagnostic performances in the identification of the pre-pubertal and post-pubertal phases, respectively [15]. Therefore, excluding those stages that are clearly related to the pre-pubertal and post-pubertal growth phases, the combination of the maturational stages of these teeth might improve the diagnostic performance for the identification of the pubertal growth spurt.

The present study was thus aimed at the determination of the diagnostic performance of the combined circumpubertal dental maturation stages of the mandibular canine and the second molar, for the identification of the growth phases of individual subjects, with particular reference to the pubertal growth spurt.

## Methods

### Study population and design

This study enrolled subjects who were seeking orthodontic treatment and who had never been treated before. Signed informed consent was obtained from the parents of the subjects prior to entry into the study, and the protocol was reviewed and approved by the local ethical committee. The following enrolment criteria were observed: (1) age between 7 and 17 years, (2) intermediate or late mixed, or early permanent phases of dentition, and (3) good general health with absence of any nutritional problems. The subjects were scheduled for enrolment at their first clinical examination, when dental panoramic radiographs and lateral cephalograms were taken. A total of 300 subjects, constituting a subset of a previous study [15], were enrolled in the study: 192 females and 108 males (mean age, 11.4 ± 2.4 years; range, 7.0 to 17.0 years).

### Assessment of individual dental maturity

Assessment of dental maturity was carried out through the calcification stages, according to the method of Demirjian et al. [16] (stages D to H), from the panoramic radiographs of the left-side mandibular teeth. Briefly, these stages are defined as:

•*Stage D*. When (1) the crown formation is complete down to the cemento-enamel junction; (2) the superior border of the pulp chamber in the uniradicular teeth has a definite curved form, with it being concave towards the cervical region; the projection of the pulp horns, if present, gives an outline shaped like the top of an umbrella; and (3) the beginning of root formation is seen, in the form of a spicule.

•*Stage E*. When (1) the walls of the pulp chamber form straight lines, the continuity of which is broken by the presence of the pulp horn, which is larger than that in the previous stage and (2) the root length is less than the crown height.

•*Stage F*. When (1) the walls of the pulp chamber form a more or less isosceles triangle, with the apex ending in a funnel shape, and (2) the root length is equal to or greater than the crown height.

•*Stage G*. When the walls of the root canal are parallel and its apical end is still partially open.

•*Stage H*. When (1) the apical end of the root canal is completely closed, and (2) the periodontal membrane has a uniform width around the root and the apex.

An experienced orthodontist (LC) who was blinded to the skeletal maturation stages assessed the dental maturity of the mandibular canines, the first and second premolars, and the second molars.

### Assessment of individual skeletal maturity

Assessment of skeletal maturity was carried out through the cervical vertebral maturation (CVM) method [2] on lateral cephalograms. This method comprises six stages (CS1 to CS6) for cervical vertebral maturation. An experienced orthodontist (GP) who was blinded to the dental maturation stages assessed the skeletal maturity of the subjects. Finally, the subjects were clustered into three groups according to their growth phases, as pre-pubertal (CS1 and CS2), pubertal (CS3 and CS4), and post-pubertal (CS5 and CS6) [8].

### Statistical analysis

For each canine and second molar, and within each dental maturation stage, the prevalence of the growth phases was calculated. To establish the clinical performance of each dental maturation stage for the diagnosis of each CVM stage, the positive likelihood ratios (LHRs) were calculated, along with the 95% confidence intervals (CIs) [17]. These positive LHRs provide estimates of how much a given dental maturation stage changes the odds of having a given growth phase. Here, a positive LHR indicates that a subject who tests positive for any clinical parameter (i.e., any dental maturation stage) has a high probability of having the given condition that needs to be diagnosed (i.e., any skeletal maturation stage). The positive LHR incorporates both the sensitivity and the specificity of the test, and it provides a direct estimate of how much a test result changes the odds of having a condition [17].

With a positive LHR ≥10 considered as the requirement for a satisfactorily diagnostic performance [18], all of the maturational stages of the individual teeth that satisfied this condition for the identification of either pre-pubertal or post-pubertal growth phases were excluded from the analysis of possible combinations of maturational stages. According to these data, the canine stages G and H and the second molar stages F and G were considered as possible combinations, for which the diagnostic performance for the identification of the growth phases was calculated. These stages were combined using either two or three stages per combination. In the case of three stages, for instance, canine stage G was combined with second molar stage F and G; similarly, canine stage H was combined with the same second molar stage F and G. The positive LHRs were retrieved for each combination, along with the 95% CIs; again, a threshold value ≥10 was used to assess a reliable diagnostic performance for the identification of growth phase on an individual basis [15,18].

The weighted kappa statistics for intra-examiner agreement were >0.88. A *p* value less than 0.05 was used for rejection of the null hypothesis.

## Results

The analyses carried out within each gender yielded generally similar results, and the data are therefore presented here as a single collective sample. The distributions of the different individual dental maturation stages according to the growth phases are shown in Table 1. The canine stages up to stage F and the second molar stages up to stage E were mostly present in the pre-pubertal growth phases; all of the other stages were distributed throughout the three growth phases, with the exception of the second molar stage H which was seen only in the pubertal and post-pubertal growth phases.

**Table 1:** Relative distributions of the individual dental maturation stages according to the growth phases

**Tooth**	**Maturation stage**	***N***	**Growth phase**		
			**Pre-pubertal (%)**	**Pubertal (%)**	**Post-pubertal (%)**
Canine	E	2	100.0	0	0
	F	80	95.0	5.0	0
	G	70	71.4	22.9	5.7
	H	148	18.9	29.7	51.4
Second molar	D	35	94.3	5.7	0
	E	76	93.4	6.6	0
	F	49	63.3	24.5	12.2
	G	84	25.0	39.3	35.7
	H	56	0	21.4	78.6

The positive LHRs for the different individual dental maturation stages for the identification of each growth phase stage are shown in Table 2. Most of these positive LHRs were ≤1.6, with values ≥10 seen only for the identification of the pre-pubertal growth phase for canine stage F (positive LHR, 17.5) and second molar stages D and E (positive LHRs, 15.2 and 13.1, respectively). Moreover, second molar stage H also yielded a positive LHR of 10.1 for identification of the post-pubertal growth phase.

**Table 2:** Positive likelihood ratios for the individual dental maturation stages for the diagnosis of growth phases

**Tooth**	**Maturation stage**	**Growth phase**		
		**Pre-pubertal**	**Pubertal**	**Post-pubertal**
Canine	E	-	-	-
	F	*17.5* (6.5 to 46.7)	0.2 (0.1 to 0.5)	-
	G	2.3 (1.4 to 3.7)	1.1 (0.7 to 1.8)	0.2 (0.1 to 0.4)
	H	0.2 (0.1 to 0.3)	1.6 (1.2 to 1.9)	2.9 (2.4 to 3.5)
Second molar	D	*15.2* (3.7 to 62.3)	0.2 (0.1 to 0.9)	-
	E	*13.1* (5.4 to 31.5)	0.3 (0.10.6)	-
	F	1.6 (0.9 to 2.7)	1.2 (0.7 to 2.2)	0.4 (0.2 to 0.9)
	G	0.3 (0.2 to 0.5)	2.4 (1.7 to 3.6)	1.5 (1.1 to 2.2)
	H	-	1.0 (0.6 to 1.8)	*10.1 *(5.7 to 18.1)

Data are presented as mean (95% confidence interval). Hyphens denote null value. Italicized values are mean positive LHRs ≥10.

The distributions of the different combined dental maturation stages according to the growth phases are shown in Table 3, including canine stages G and H, and second molar stages F and G. Generally, none of the combined dental maturation stages, either as two or three stages combined, showed any clear prevalence among any of the different growth phases. In the best cases, canine stage G in combination with second molar stage F, or both stages F and G, were seen mostly for the pre-pubertal and pubertal growth phases (93% and 90.9%, respectively). Similarly, 89.1% of canine stages G and H in combination with second molar stage F was seen in the pre-pubertal and pubertal growth phases.

**Table 3:** Relative distributions of the combined dental maturation stages according to the growth phases

**Tooth and maturation stage**	***N***	**Growth phase**		
		**Pre-pubertal (%)**	**Pubertal (%)**	**Post-pubertal (%)**
*Combination of two stages*				
Canine G with second molar F	32	68.8	25.0	6.3
Canine G with second molar G	12	41.7	41.7	16.7
Canine H with second molar F	17	52.9	23.5	23.5
Canine H with second molar G	72	22.2	38.9	38.9
*Combination of three stages*				
Canine G with second molar F/G	44	61.4	29.5	9.1
Canine H with second molar F/G	89	28.1	36.0	36.0
Canine G/H with second molar F	46	60.9	28.3	10.9
Canine G/H with second molar G	87	27.6	36.8	35.6

The cases for the canine stage up to stage F or the second molar stage up to stage E, and those for the second molar stage from stage H, which showed positive LHRs ≥10 for the diagnosis of the pre-pubertal and post-pubertal growth phases, respectively, are not shown.

The positive LHRs for the different combined dental maturation stages for the identification of each growth phase stage are shown in Table 4. All of these positive LHRs were ≤2.6, irrespective of the combinations of two or three maturational stages. The greatest positive LHR of 2.6, with 95% CI up to 8.0, was seen for the combined stages G of both canine and second molar for the identification of the pubertal growth phase. Generally, slightly greater values were seen for the pubertal growth phase as compared to the pre-pubertal and post-pubertal growth phases.

**Table 4:** Positive likelihood ratios for the combined dental maturation stages for the diagnosis of growth phases

**Tooth and maturation stage**	**Growth phase positive**		
	**Pre-pubertal**	**Pubertal**	**Post-pubertal**
*Combination of two stages*			
Canine G with second molar F	2.0 (1.0 to 4.1)	1.2 (0.6 to 2.6)	0.2 (0.1 to 0.8)
Canine G with second molar G	0.7 (0.2 to 2.0)	2.6 (0.9 to 8.0)	0.6 (0.1 to 2.5)
Canine H with second molar F	1.0 (0.4 to 2.6)	1.1 (0.4 to 3.4)	0.8 (0.3 to 2.6)
Canine H with second molar G	0.3 (0.2 to 0.4)	2.3 (1.6 to 3.4)	1.8 (1.2 to 2.6)
*Combination of three stages*			
Canine G with second molar F/G	1.5 (0.8 to 2.6)	1.5 (0.9 to 2.8)	0.3 (0.1 to 0.7)
Canine H with second molar F/G	0.4 (0.2 to 0.5)	2.1 (1.5 to 2.9)	1.5 (1.1 to 2.2)
Canine G/H with second molar F	1.4 (0.8 to 2.5)	1.5 (0.8 to 2.6)	0.3 (0.1 to 0.8)
Canine G/H with second molar G	0.4 (0.2 to 0.5)	2.1 (1.5 to 3.0)	1.5 (1.0 to 2.2)

Data are presented as mean (95% confidence interval). The cases for the canine stages up to stage F or the second molar stages up to stage E, and those for the second molar stages from stage H, which showed positive LHRs ≥10 for the diagnosis of the pre-pubertal and post-pubertal growth phases, respectively, are not shown.

## Discussion

The present study investigated the diagnostic performance of the circumpubertal combined maturation stages of the mandibular canine and second molar for the identification of the growth phases. The data showed that when the stages that are clearly related to the pre-pubertal and post-pubertal growth phases are excluded, all of the combinations of two or three of the remaining dental maturity stages showed limited clinical usefulness for the identification of individual skeletal maturity.

Dental maturity assessment offers the advantage of being a simple procedure that can be carried out on panoramic radiographs that are routinely used for different purposes, and intra-oral radiographs can be taken with minimal irradiation to the patient. Moreover, the method described by Demirjian et al. [16] has the advantage of being little influenced by dimensional distortions that might be associated with panoramic radiographs. For this reason, several investigations [10-15] have been focused on such indicators of skeletal maturity. All of these studies have reported considerably high correlation coefficients between the dental maturational stages and the skeletal maturation/growth phases. However, a high correlation coefficient alone does not prove that any dental maturation stage has a satisfactory performance for the diagnostic identification of the skeletal maturation/growth phases on an individual basis.

In the present study, only the dental stages F for the canine and D and E for the second molar yielded any satisfactory diagnostic performance in the identification of the pre-pubertal growth phase. Similarly, only the second molar stage H was shown to be a reliable indicator of the post-pubertal growth phase, although the positive LHR was just above the threshold, at 10.1, with 95% CI ranging from 5.7 to 18.1 (Tables 1 and 2). None of the dental stages for either tooth were reliable in the identification of the pubertal growth phase, which were also the most important developmental stages to be identified in treatment planning, as for instance, in the case of skeletal class II malocclusion [2]. Irrespective of ethnicity, previous studies have reported close relationships between mandibular canine stages G [10] and F, [13] or between the two [12], and the pubertal growth spurt. Similar results were reported in more recent investigations [19,20]. Of interest, a recent study [14] also showed increased prevalence of the mandibular second molar stage H with the post-pubertal growth phase.

However, all of these investigations [10-14,19,20] were limited to analyses of the distributions of the dental maturation stages according to the skeletal maturation stages, and they lacked any specific diagnostic performance analysis. In contrast, the present data are in line with those of the only previous study on the diagnostic performance of individual mandibular teeth [15].

With the evidence that the canine stages up to stage F and the second molar stages up to stage E were mostly pre-pubertal, and the second molar stage H was mostly post-pubertal, these stages were excluded from the analysis of the diagnostic performance of the combined dental maturational stages. The combinations analyzed, thus, included the canine stages G and H, and the second molar stages F and G. Moreover, the possible combinations between these maturational stages of the mandibular canine and second molar were clustered using one stage per tooth (two maturational stages) or more (three maturational stages) for a total of eight clusters (Table 4). As shown by the positive LHRs, none of the combinations investigated showed any satisfactory level of diagnostic performance. Although the positive LHRs for the identification of the pubertal growth phase (Table 4) were slightly greater than those obtained for the individual maturational stages (Table 2), these coefficients remained far from the desired threshold, with the best value of 2.6 for combination of the canine and the second molar stage G. Therefore, although an improvement in the diagnostic performance was seen for the identification of the pubertal growth phase, this was not sufficient to propose such combined dental maturation as a reliable indicator of the pubertal growth spurt.

The diagnostic performance of the dental maturity for the identification of specific stages of skeletal maturity will thus be limited. The developmental status of the mandibular canine and the second molar might only be useful in the diagnosis of the pre-pubertal and post-pubertal growth phases. The combination of the maturational stages of these two teeth does not increase the diagnostic performance in the identification of any of the growth phases. Therefore, precise information about the timing of the onset of the growth spurt, with the relevant clinical implications in the treatment of skeletal class II subjects, is not provided by these dental indices.

## Conclusions

With the above results, we have the following conclusions: (1) The diagnostic performance of dental maturity when considering individual teeth is reliable only for the identification of the pre-pubertal and post-pubertal growth phases. (2) The combination of maturational stages of the mandibular canines and the second molars provides a slightly improved diagnostic performance for the identification of the pubertal growth phase, although this remains unsatisfactory. (3) Dental maturation of either individual or combined teeth has little role in assessing the timing for treatments that are required to be performed during the pubertal growth spurt.

## Competing interests

The authors declare that they have no competing interests.

## Authors’ contributions

GP, conceived the study, selected cases and did the statistical analysis; RDL, supervised the team; LC, collected data and wrote the manuscript with RDL. All authors read and approved the final manuscript.
